# Double-diaphragm forming of highly aligned short-fibre preforms for complex composite parts

**DOI:** 10.1007/s12289-025-01952-1

**Published:** 2025-10-06

**Authors:** Tharan Gordon, Ogun Yavuz, Bohao Zhang, Xiaochuan Sun, Ian Hamerton, Marco L. Longana, Stephen R. Hallett, Jonathan P.-H. Belnoue, Byung Chul Kim

**Affiliations:** 1 Bristol Composite Institutes, School of Civil, Aerospace, and Design Engineering, University of Bristol, Queen’s Building, University Walk, Bristol, BS8 1TR UK; 2 Department of Chemistry, Materials, and Chemical Engineering “Giulio Natta”, Politecnico di Milano, Milan, 20133 Italy

**Keywords:** Composite forming, Aligned discontinuous fibres, Defects, Process modelling

## Abstract

Forming small to medium composite parts with complex geometries presents significant challenges to engineers, primarily due to material-induced, in-process defects such as fibre bridging and wrinkling, leading to poor mould conformity. These issues are characteristic of continuous fibre preforms and the inextensibility of the fibres. HiPerDiF (High Performance Discontinuous Fibre) technology is a novel manufacturing technique to produce high-performance, aligned discontinuous fibre pre-preg materials. This study investigates the forming characteristics of prepreg manufactured using the HiPerDiF method, highlighting its viability for complex part manufacture where mould conformity is critical. Additionally, a previously developed finite element (FE) model, able to predict the behaviour discontinuous fibre preforms during double diaphragm forming (DDF), was used to obtain insights into the experimentally observed material deformation. The results demonstrated the advantage of the enhanced formability of the HiPerDiF preform, owing to its stretchability in the double-diaphragm vacuum forming process. The FE simulations were shown to be a powerful tool to gain understanding of preforms deformation and thickness variation which are otherwise difficult to measure experimentally.

**Graphical abstract:**

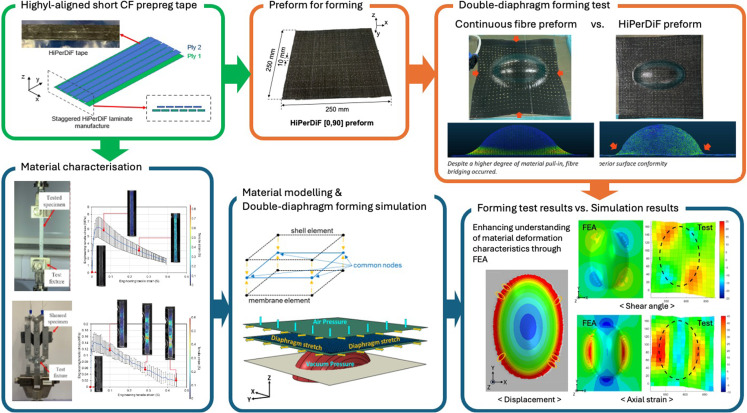

## Introduction

Diaphragm forming is an attractive and scalable manufacturing method suitable for the high-volume production of geometrically complex 3D components. In particular, vacuum-assisted Double Diaphragm Forming (DDF) is a popular method offering enhanced stability of the preform during forming, which is achieved by securely holding a complete stack of plies or fabric between two deformable diaphragms through the application of a vacuum between them. This sandwiched assembly is then drawn over a rigid mould, either male or female, utilising the pressure difference between the top and bottom of the assembly.

In comparison to stamp forming, where two rigid mould surfaces are employed, DDF can offer significant advantages, including lower capital investment and reduced processing times attributed to the minimised thermal masses of the mould. However, when forming composites over highly complex shapes, a key challenge in its implementation arises from the generation of undesired geometrical defects. This challenge stems low forming pressure during the drawing process over the mould, coupled with the limited formability of the preform-diaphragm assembly. As such, in DDF there is the potential for thickness variations in the preform as it undergoes shearing, as well as a higher degree of wrinkling and/or bridging [1]. Such deviations from geometric tolerances and defects can, in turn, compromise the structural performance of the final components.

Wrinkling in DDF has been demonstrated to be multi-factorial, influenced by both ply and preform level properties as well as processing parameters [1, 2]. Thompson et al. [3] identified multiple key parameters influencing the forming behaviour, including fibre orientation, inter-ply friction, and bending stiffness, all of which contribute to the onset, shape, and severity of wrinkles. In terms of processing parameters, the tension applied in the plane of the fabric also affected its ability to conform to the mould geometry, with proper control helping to minimise defect introduction. In a detailed review, Boisse et al. [4], have highlighted that the bending stiffness of the preform, whilst typically very low, plays an important role determining the size of the wrinkles formed. Importantly, the relative inextensibility of fibres leads to bending response being dominated by inter-ply slippage, which is a phenomenon particularly critical for preforms with continuous fibres aligned more towards the bending direction.

Much work has been undertaken to optimise various parameters for enhancing the formability of preforms in DDF, such as the stacking sequence investigations mentioned above, or the incorporation of additional mould elements to selectively induce localised in-plane stretching to the ply stack and diaphragms during forming [5]. In a similar conceptual vein, a recent article [6] explores the development of a surrogate model, designed to rapidly predict the relationship between mould geometry and preform wrinkling patterns. This type of approach however, whilst promising for minimising defect introduction, still ultimately imposes design constraints based on material-based limitations.

A particularly promising research avenue lies in exploring material systems with enhanced deformability. Highly aligned, discontinuous fibre prepregs are a class of material that can provide comparable material properties to continuous fibre composites after curing, despite their discontinuities [7], provided the fibre length and alignment are sufficient for effective load transfer between adjacent fibres. These materials are also known to offer improved formability, attributed to the ability of the fibres to slide past one another within the material [8]. Historically, their limited industrial use stemmed from the low productivity of manufacturing processes, with the fibre alignment stage being particularly inefficient. However, recent advancements in scalable production, enabled by the use of water as a medium for fibre alignment, have revitalised interest in both the manufacture and modelling of these materials, thereby supporting the industrial uptake. Cender et al. have developed a material model for a thermoplastic based highly aligned discontinuous fibre materials and correlated the model with the tensile test results of a PEI matrix material [9]. Morris et al. carried out double-diaphragm forming of a PEKK based material on a convex mould with a shape of half a cylindrical hemisphere and proposed a forming limit diagram for that material [10]. Tomlin et al. characterised a B-staged epoxy-based material and proposed a corresponding forming limit diagram based on its tensile test result [11].

The water-based fibre alignment process was originated from the High-Performance Discontinuous Fibre (HiPerDiF) technology^1^ with the capability of aligning the fibres along the direction of production, which enables the large-scale production of such materials. It has been shown that a carbon-epoxy HiPerDiF tape with 3 mm long aligned fibres (55% V_f_) achieved a tensile strength of ~ 1500 MPa, and a modulus of 115 GPa [12]. However, HiPerDiF tape is still an emergent material, and there is limited knowhow regarding its optimal processing for DDF of complex 3D shapes. This contrasts with dry fabric materials and continuous fibre prepregs, where numerous authors have made significant progress in simulating the DDF forming process [13–15].

This study explored the potential of using a highly aligned, discontinuous carbon fibre prepreg in DDF for the high-quality production of small and highly complex composite parts that are challenging to produce with continuous fibre prepregs. In the initial phase, highly aligned short fibre preforms were produced using the HiPerDiF process, and material characterisation was conducted to facilitate the development of a suitable material model. Subsequently, the material model, which was initially developed by Thompson et al. [3] and revised by Yavuz et al. [16] to capture the material behaviour in the low tensile strain region, was further extended in the present study to account for higher tensile strains as well as shear deformation. This enhanced model was then integrated into a finite element forming simulation to predict large deformations of the HiPerDiF material across a doubly curved, complex geometry. Finally, DDF tests were conducted, and laser 3D profile scans were used to capture any wrinkles or local deformations in the preform.

## Methodology

### Materials characterisation

#### Specimen manufacture

The material used in this study was a unidirectional HiPerDiF tape, manufactured using the third generation HiPerDiF machine, previously reported in [17].

**Fig. 1:**
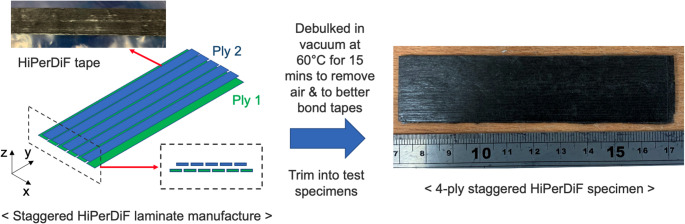
Specimen preparation for tension, shear and bending tests with the HiPerDiF tapes

To inform the numerical simulation work, the material behaviours of the uncured HiPerDiF tape were experimentally characterised in both tension and shear. Figure 1 shows the specimen preparation procedure for both tests. Firstly, a 25 mm-wide, dry, single-ply tape with a fibre areal weight of 32.1 gsm was first produced from 3 mm long carbon fibres (Tenax-A HT C123, Teijin Ltd., Tokyo, Japan), and was impregnated with a B-staged epoxy resin film (MTM49-3, Syensqo, UK) into a 25 mm-wide, aligned, discontinuous fibre prepreg tape. The fibre volume fraction (V_f_) of this single-ply ply thickness HiPerDiF tape was approximately 27%, with an average areal weight of 109.1 gsm. Then, four plies of the material were subsequently stacked in a staggered configuration (see Fig. 1). The preform was then debulked at 60 °C for 15 min to remove trapped air bubbles and to improve the adhesion between plies. After this, tensile specimens of 160 mm in length and 10 mm in width and in-plane shear specimens of 100 mm in length and 25 mm in width were prepared from these resulting preforms. The fibre direction was along the longer side of the specimens.

#### Test method

**Fig. 2:**
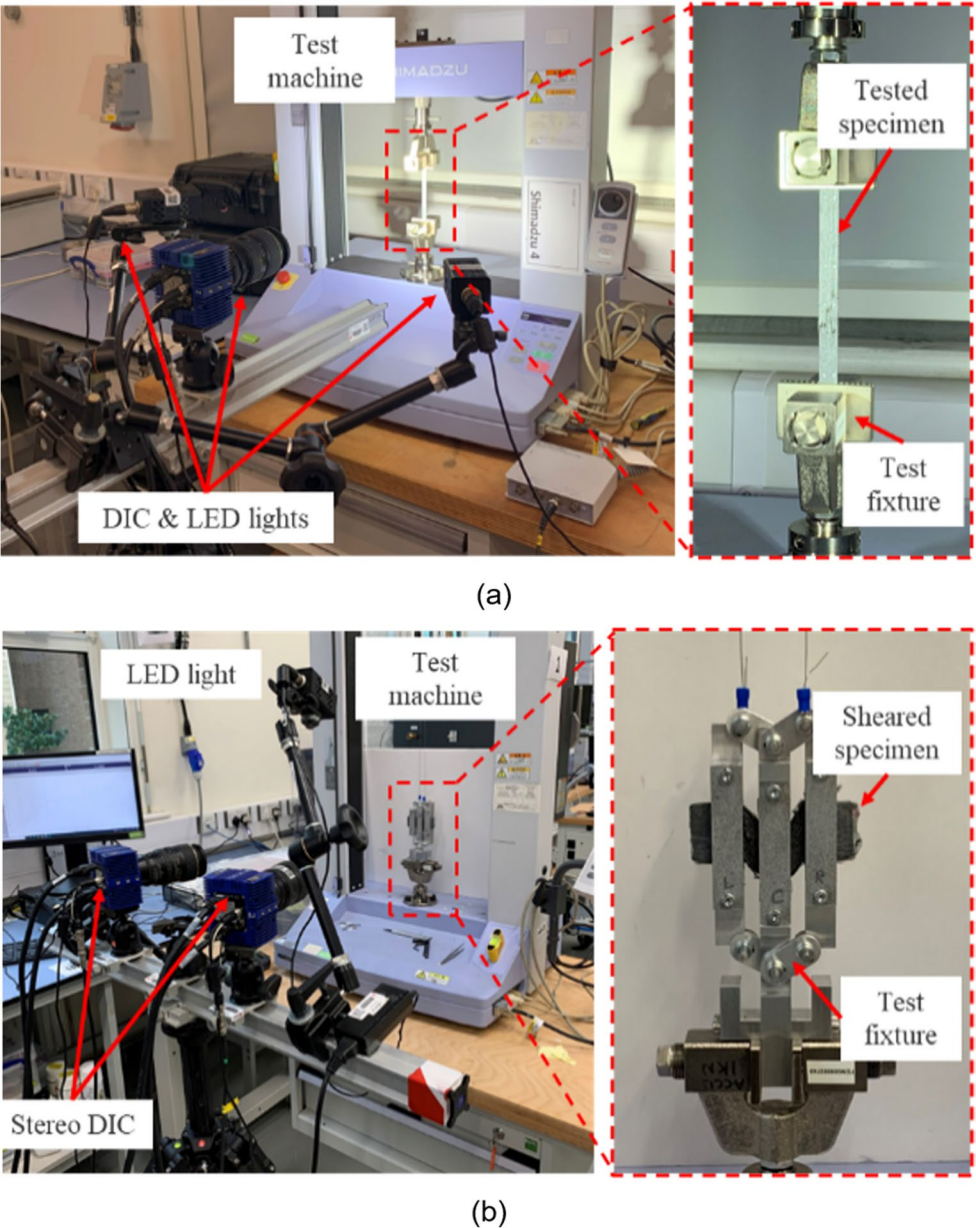
Test setups of (**a**) the tensile test and (**b**) the in-plane shear test

Figure 2 shows the setup of the tensile and shear tests of the uncured specimens prepared following the procedures described above. The tensile test was conducted on an electro-mechanical universal test machine (Shimadzu, JP) fitted with a 1 kN loadcell. Three specimens were tested at each of room temperature and 50 °C at constant crosshead speed of 5 mm/min until failure. A heater gun with an integrated airflow and temperature controller was used to regulate the temperature in the test area of the specimen, and a thermal camera (FLIR T650, US) was used to monitor specimen surface temperature. Digital image correlation (DIC) device (LaVision, DE) with a 16 mega-pixel camera was used to measure tensile strain over the gauge length of 100 mm on the specimens.

The shear test was performed using a modified picture frame test rig [18] on the same test machine, with a 100 N loadcell. The shear specimen, which was horizontally oriented, was clamped with three aluminium blocks, while leaving 10 mm shearing gaps in between the blocks. The specimen was sheared in-plane by pulling the side blocks, which were mechanically linked with the central fixed clamping block, upwards. Three shear tests were performed at each of room temperature and 50 °C at the constant shear strain rate of 0.03 rad/s (approx. 1.72°/s) until a global shear angle of 45° was achieved. Stereo DIC with two 16MP cameras was used to capture in-plane shear strain development over the sheared regions in between the clamping blocks.

### Forming experiments

#### Preform and mould manufacture

**Fig. 3:**
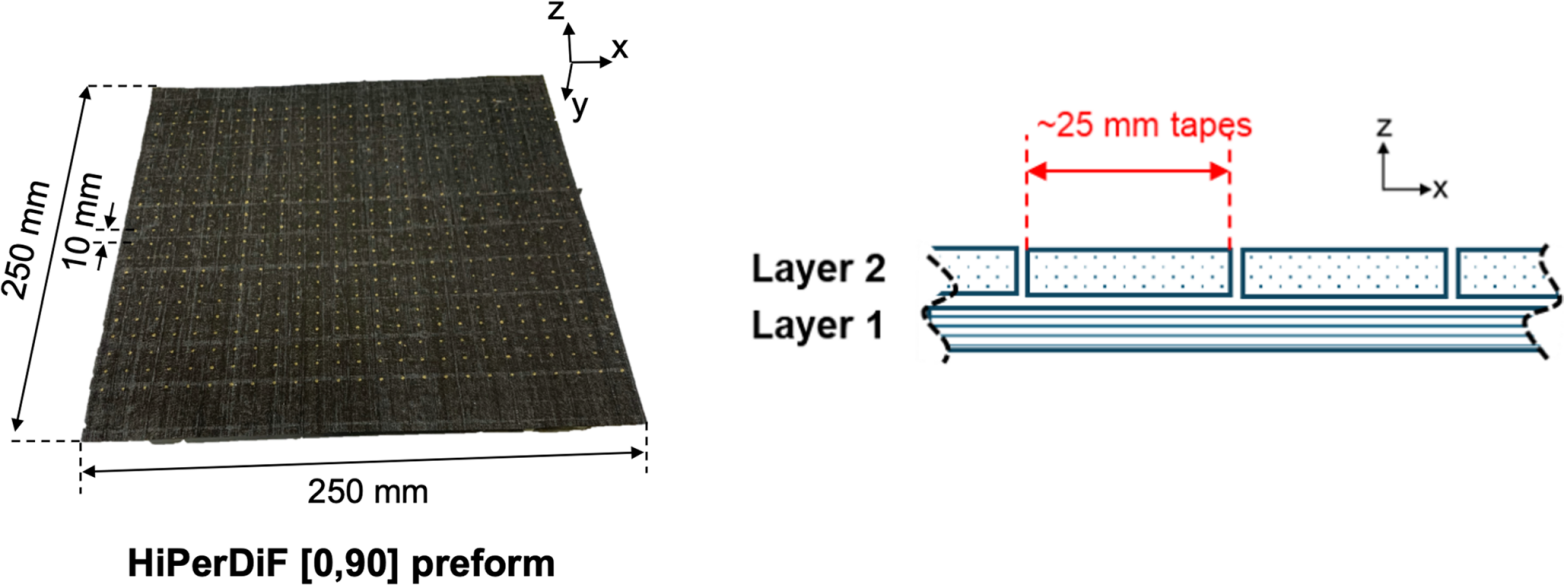
HiPerDiF preform with a dot grid pattern on its surface, and the stacking sequence

A square, cross-ply preform with the dimensions of 250 mm x 250 mm was manufactured *via* hand layup, with each ply prepared by arranging single strips of the 25 mm wide HiPerDiF tapes described in “Specimen manufacture” section side by side. The stacking sequence used was [0/90], and once stacked the whole preform was debulked in the same manner as the materials characterisation specimens. A grid of dots spaced regularly at 10 mm intervals was then subsequently applied to the preform surface, for measuring the tensile and shear deformation during forming, as shown in Fig. 3.

**Fig. 4:**
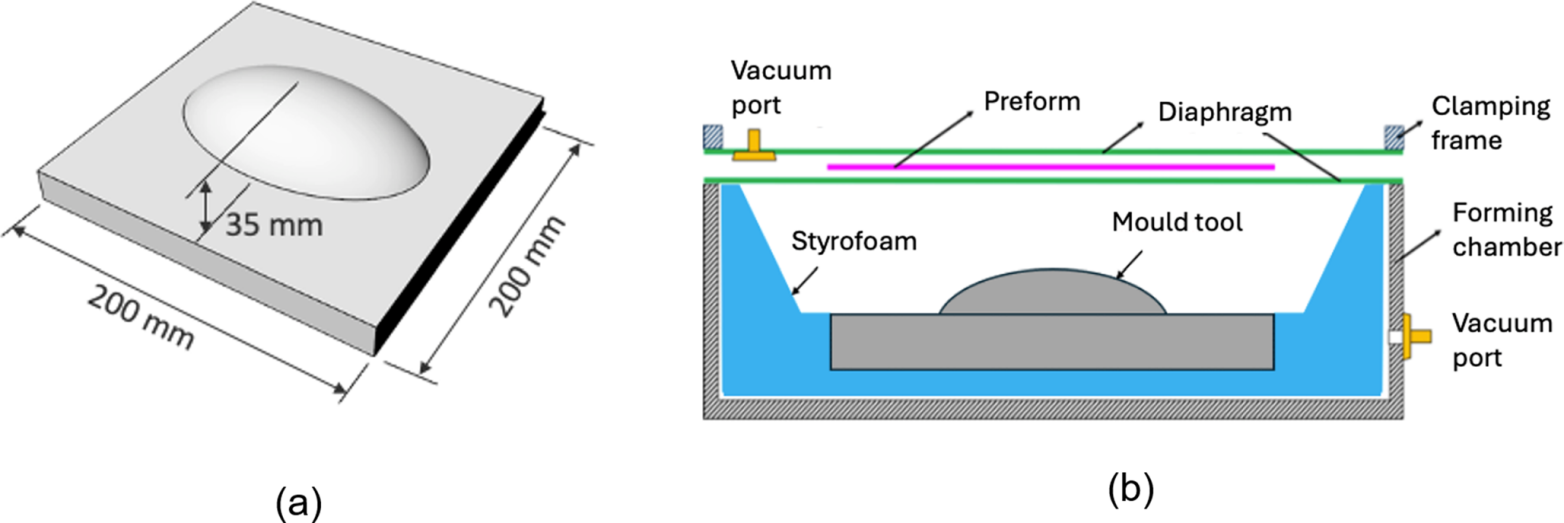
**a** Doubly curved mould tool, and (**b**) double-diaphragm forming rig

The preform thickness was measured as 0.25 mm, averaged from multiple micrometer measurements at different locations, and the mass was measured to be 14.1 g. For the mould surface, a doubly curved convex mould with a positive Gaussian curvature was machined from billet aluminium *via* CNC, with the dimensions as shown in Fig. 4a.

Double Diaphragm test.

The double diaphragm forming test rig, as described in previous work [15], consisted of an open-top enclosure to accommodate the mould, sloping Styrofoam internal walls and a frame for manual clamping of the two diaphragms that hold the preform in between (see Fig. 4b). The order of forming operations was as follows:

i. The preform was first placed between two thin highly stretchable diaphragms (Stretchlon 200, Airtech Advanced Materials UK Ltd., UK) whose thickness and elongation limit are 38 μm and about 500%, respectively. The preform was aligned in such a way the fibres are in line with the principal curvature directions of the mould, and a small amount of pre-tension was applied to the preform/diaphragms assembly around its perimeter when clamping its edges on the top of the frame. A vacuum was applied between these two membranes to hold the preform securely in position.
ii. An electrical heating fan was used to bring the suspended preform to the desired temperature for the process (50 ± 5 °C), which was measured using a pair of thermocouples attached to the diaphragm surfaces. After reaching the target temperature range, the preform was held at that temperature for 45 s, to ensure that the preform temperature through its thickness reached an approximate equilibrium.
iii. The enclosure was then subsequently evacuated to complete the forming in approximately 60 s, by using a vacuum pump with a flow regulator. The preform temperature during this stage was monitored to maintain it within the target range.
iv. Once the forming was completed, the temperature was held for a further 45 s and vacuum was maintained for further measurements to be taken.

#### Formability evaluation

Given the curved and reflective surface of the diaphragm, alongside the large deformations experienced during forming, it was a considerable challenge to use traditional 3D strain measurement techniques such as video gauge or Digital Image Correlation (DIC), due to loss (during forming) of the ‘speckle’ pattern on the diaphragm.

**Fig. 5:**
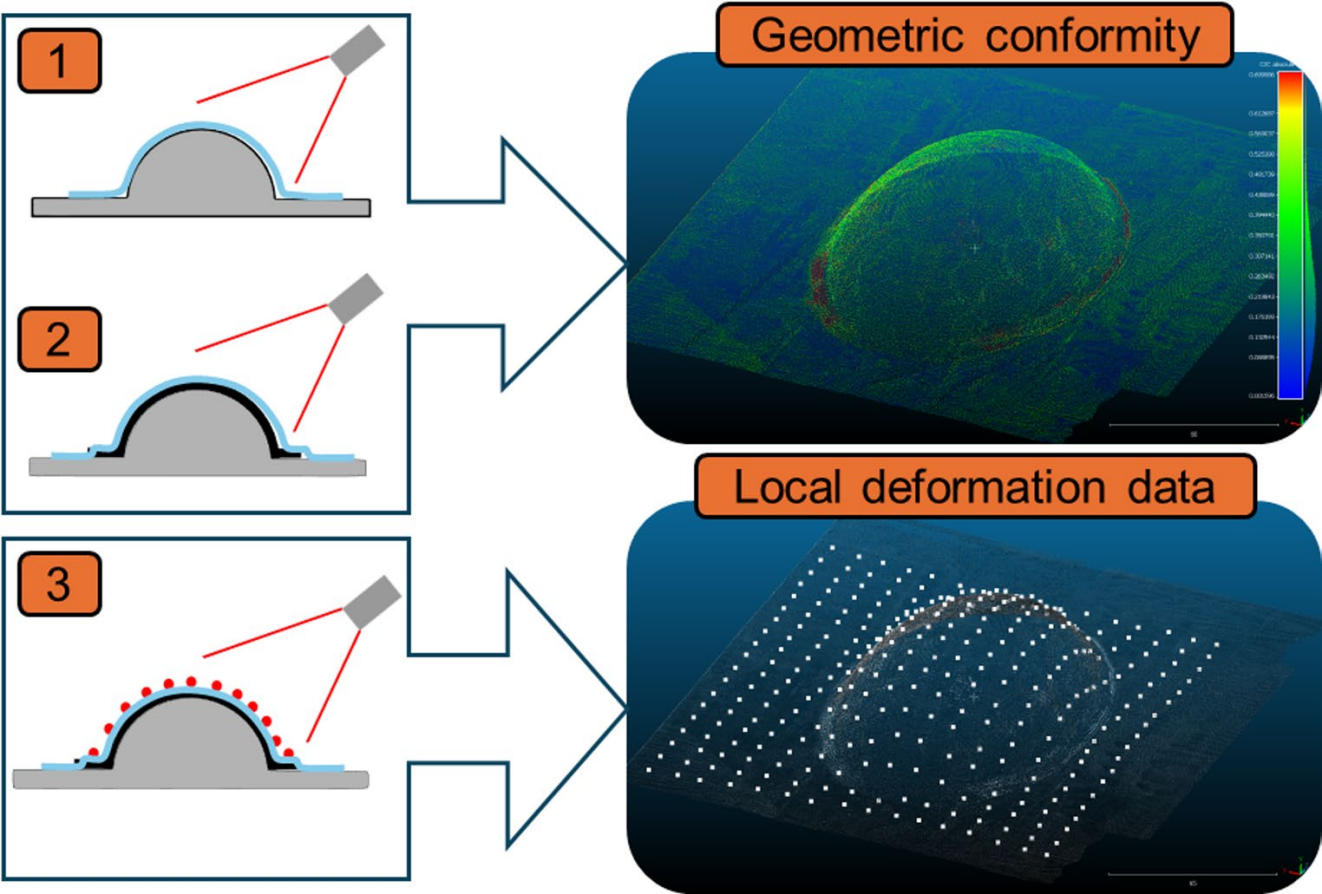
Schematic showing laser scanning procedure for quantitative evaluation of forming quality

To measure the shape of the as-formed specimens, a laser surface profile scanning method was used, as outlined schematically in Fig. 5. Firstly, the mould was scanned in the absence of the preforms (with empty diaphragms), to prepare a reference geometry. After forming the mould was then scanned again, with the preform sandwiched in between the diaphragms. Using these two data sets, the geometric conformity of the preform to the target was assessed. As shown in step 3 of Fig. 5, a small hemispherical marker with a 1 mm diameter was then placed manually on top of the diaphragm at each dot location previously marked on the preforms, which was visible through the transparent diaphragm. The preform, with the three-dimensional dot markers included was then scanned a final time.

**Fig. 6:**
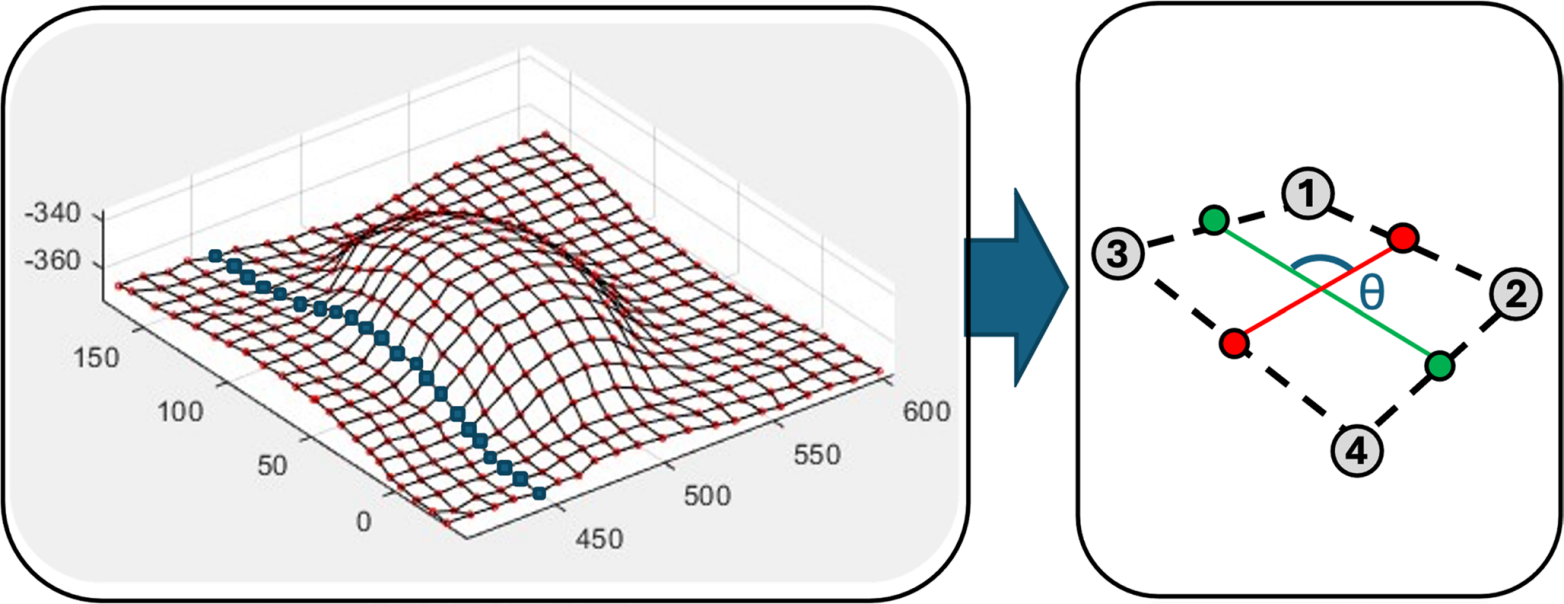
Mesh derived from the laser scanning method and a schematic showing the variables used to calculate the preform strains for each cell

Once this final scan had been taken, the point cloud surface without the markers (obtained during Step 2 in Fig. 5) was subtracted from that with the markers (obtained during Step 3 in Fig. 5), resulting in small point cloud areas representing the individual hemi-spherical markers. To convert this resulting dataset into a mesh for later strain calculations, the centroid of each of these clouds was calculated with a custom written MATLAB code using k-means clustering [19]. This method of clustering assigns a number of randomly seeded cluster centroids (k) and partitions the 3D marker data into clusters by iteratively assigning markers to the nearest centroid and then updating centroids to minimise the within-cluster sum of squared distances. In the present work, k was chosen to equal the known number of marker points, and initialised as a uniform grid, to aid in convergence. The final result of this clustering was a series of single points representing the centroid of each 3D marker point cloud – which was used to establish a mesh as shown in Fig. 6.

To calculate an approximate local strain distribution resulting from the forming process, the geometry of this mesh was compared to the known undeformed original grid, of equally spaced dots on the preform. To calculate the shear angle, the vectors representing the mid-lines of each ‘cell’ (shown as red and green lines in Fig. 6) were used for each element. The calculated change in the angle between these lines (*θ* in the figure), from the initial known value of 90° was thus reported cell-wise. A similar procedure was conducted using the Euclidean norm of the vectors describing the red and green lines in Fig. 6 (for the two different orientations, respectively), which could then be compared to the initial grid spacing of the dots (which was 10 mm as shown in Fig. 3), to give an estimate of the strains in both directions. It should be noted that the resulting strains are local and element-wise, and thus the approximate axial strains reported are not in the global coordinate system.

### Forming simulation

#### Material modelling

**Fig. 7:**
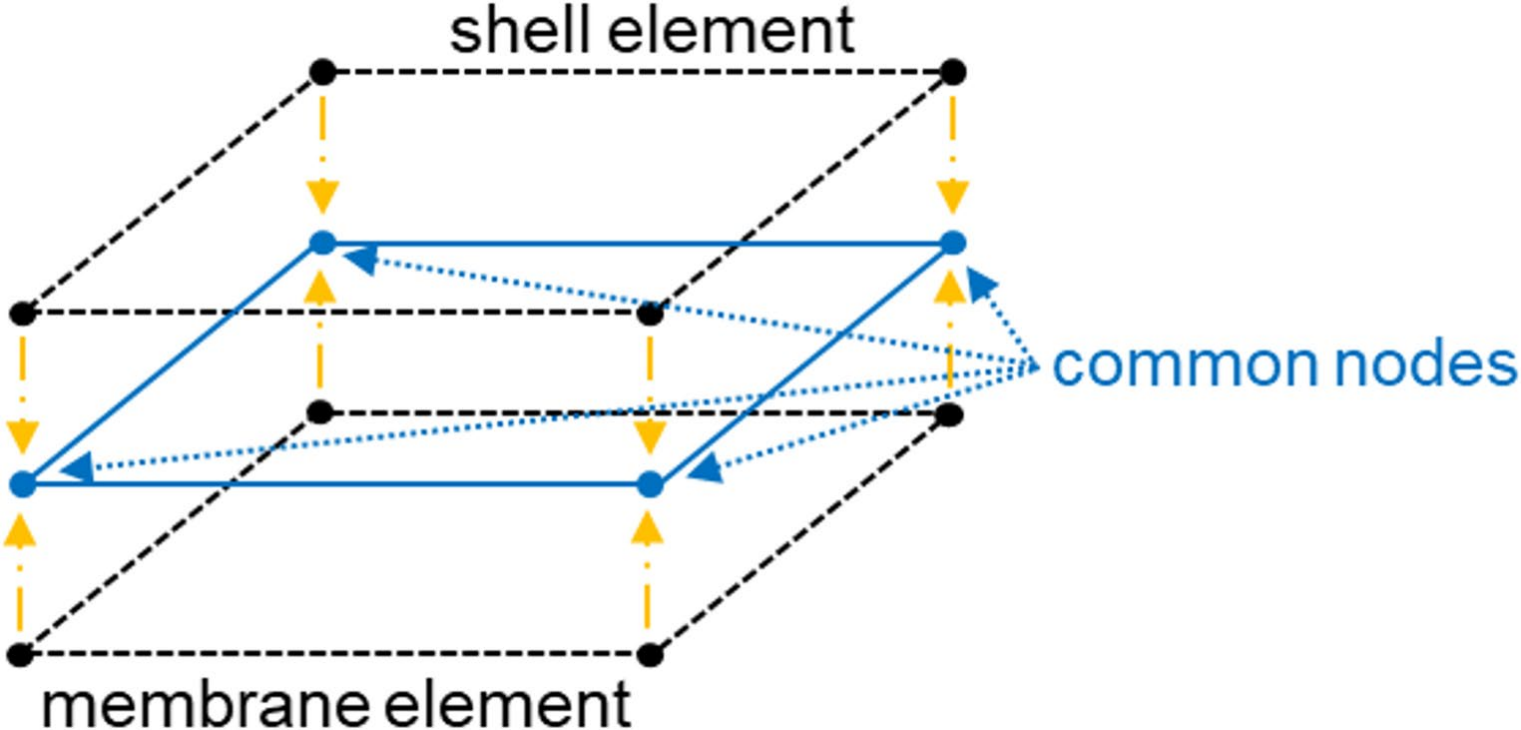
A schematic of the hybrid element comprising shell and membrane elements used in ABAQUS [16]

A numerical simulation of the double diaphragm forming experiments was conducted using the ABAQUS/explicit solver and a version of the user-defined material model developed by Thompson et al. [3], revised by Yavuz et al. [16]. In [3], shell (S4R in Abaqus) and membrane elements (M3D4R in Abaqus) are superimposed to represent and decouple out-of-plane bending and in-plane material properties of the preform, as shown in Fig. 7. However, in this study no bending test was conducted. The tensile model was therefore implemented within the shell elements only, under the assumption of Cauchy mechanics. This implies a direct coupling between tensile and bending behaviour. While this is a simplification, for aligned discontinuous fibre prepreg materials this assumption could be less prejudicial than for traditional continuous fibre prepregs. A material model was implemented for simulating the shear and tensile responses of the 2D preform, based on the data obtained from the material characterisation tests (details outlined in Materials characterisation section). Whilst the original model was designed to simulate the forming of continuous fibre composites, the revised version can be used for simulating discontinuous material, utilising an enhanced tensile micromechanical model, and a shear micromechanical model, both developed by Yavuz et al. [16]. The details of the rate equation are provided in Appendix (A.1). These material models are based on an analytical model that incorporates the assumed microstructural characteristics of the tape and the rheological properties of the resin. They were subsequently integrated into Abaqus/Explicit using a VUMAT subroutine using the following input parameters: fibre length (*L*) = 3 mm, fibre diameter (𝐷) = 7 μm, fibre volume fraction (𝑓) = 0.27, fibre overlap length (ẟ) = 1.5 mm, fibre volume fraction parameter (𝐾) = 2.64 [16].

#### Double-diaphragm forming model

**Fig. 8:**
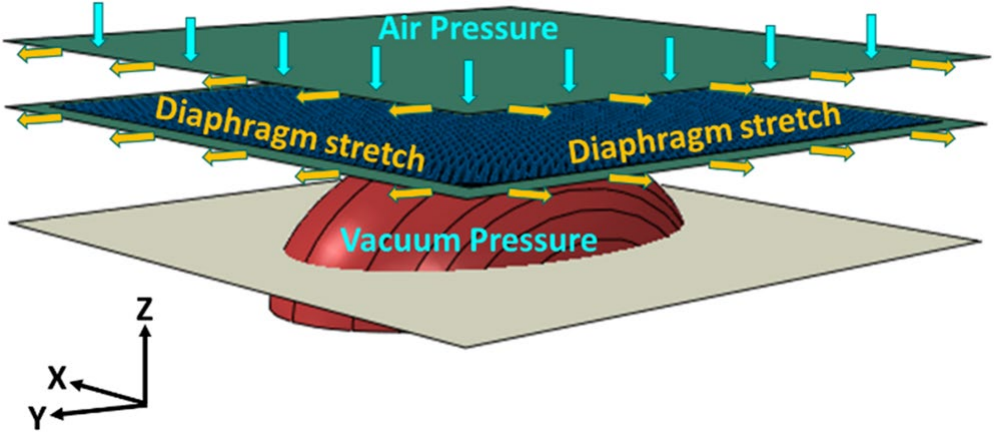
A schematic of the DDF (Double Diaphragm forming) model and boundary conditions

To analyse the forming behaviour of the HiPerDiF preforms, the DDF was simulated using ABAQUS. Each layer (which are 0° and 90°) was modelled separately by using the combination of shell and membrane elements as described above. The simulation started with applying surface pressure to both upper and lower diaphragms in opposite directions. After the diaphragms and preform assembly became stable, then the pressure acting on the lower diaphragm was gradually reduced so that the assembly was formed onto the mould shown Fig. 8. The flat and doubly-curved surfaces of the forming mould were modelled as analytically rigid, while the diaphragms were modelled as an isotropic material with Young’s modulus of 40 MPa and Poisson’s ratios of 0.3. Simplified Coulombic frictional contact was found effective in simulating prepreg/prepreg and prepreg/mould interactions. The friction coefficients for diaphragm-to-diaphragm, diaphragm-to-prepreg, prepreg-to-prepreg interfaces were set to 0.8, while the coefficient for the diaphragm-to-mould was 0.3 [20]. To replicate the initial condition of the diaphragms, which were slightly tensioned by the clamps, the simulation applied a tensioning displacement. The diaphragm was stretched by 40 mm in both directions along the x-axis and by 20 mm in both directions along the y-axis.

## Results and discussion

### Materials characterisation

**Fig. 9:**
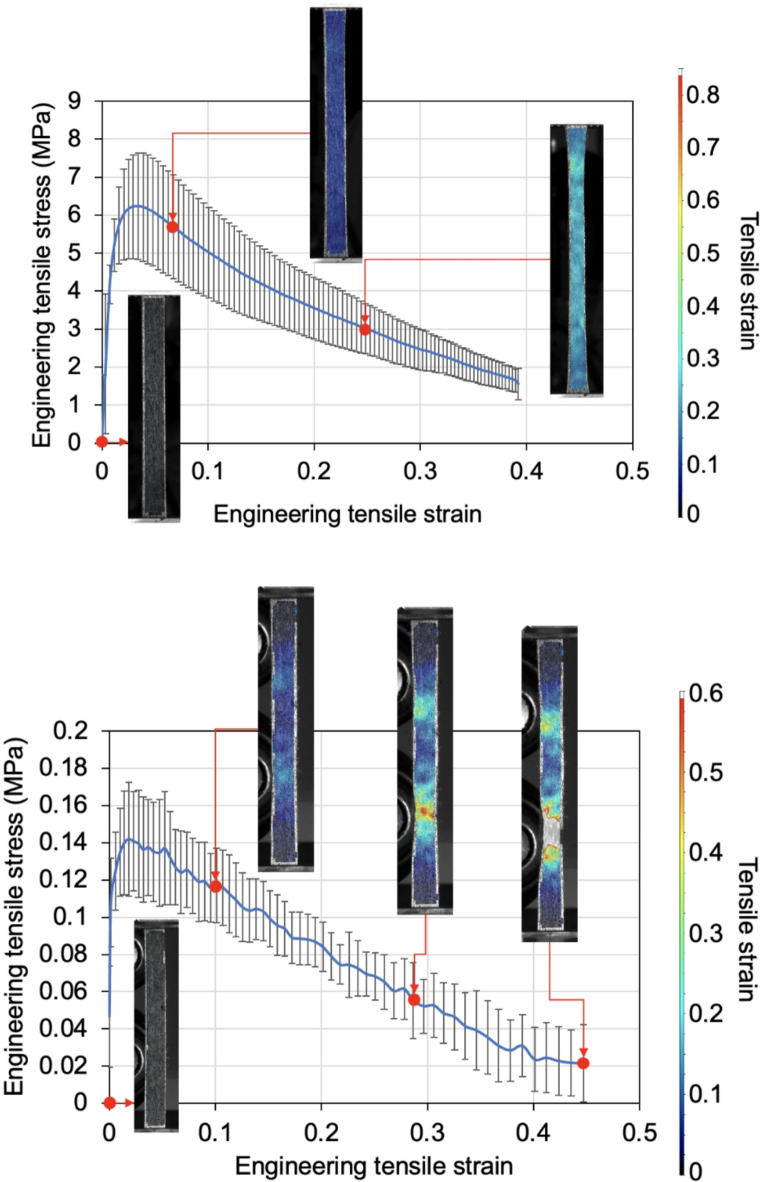
Average engineering tensile stress-strain curves of HiPerDiF specimens at (top) room temperature and (bottom) 50 °C

Figure 9 shows the average engineering tensile stress-strain plots of the specimens at room temperature and 50 °C. The material exhibited a strain-softening behaviour due to the slippage between aligned short fibres within the.

viscous matrix. This softening behaviour has been reported on uncured fibre-reinforced sheet moulding compounds in tension [21]. A large standard deviation at room temperature and 50 °C was captured, due to the variability in fibre alignment and resin distribution of the specimens. The distribution of the tensile strain during testing, as shown in the inset images in the graphs, was reasonably uniform across the gauge length initially. This tensile strain generates shear stress within the matrix, particularly between the fibres. Up to a maximum load, this shear stress contributes to an increase in the tensile stress of the specimen.

However, beyond a certain point, the fibres begin to slide over each other, reducing load transfer until total separation occurs. This complete separation is observed at approximately 0.5 tensile strain, measured locally for both room temperature and 50 °C specimens.

**Fig. 10:**
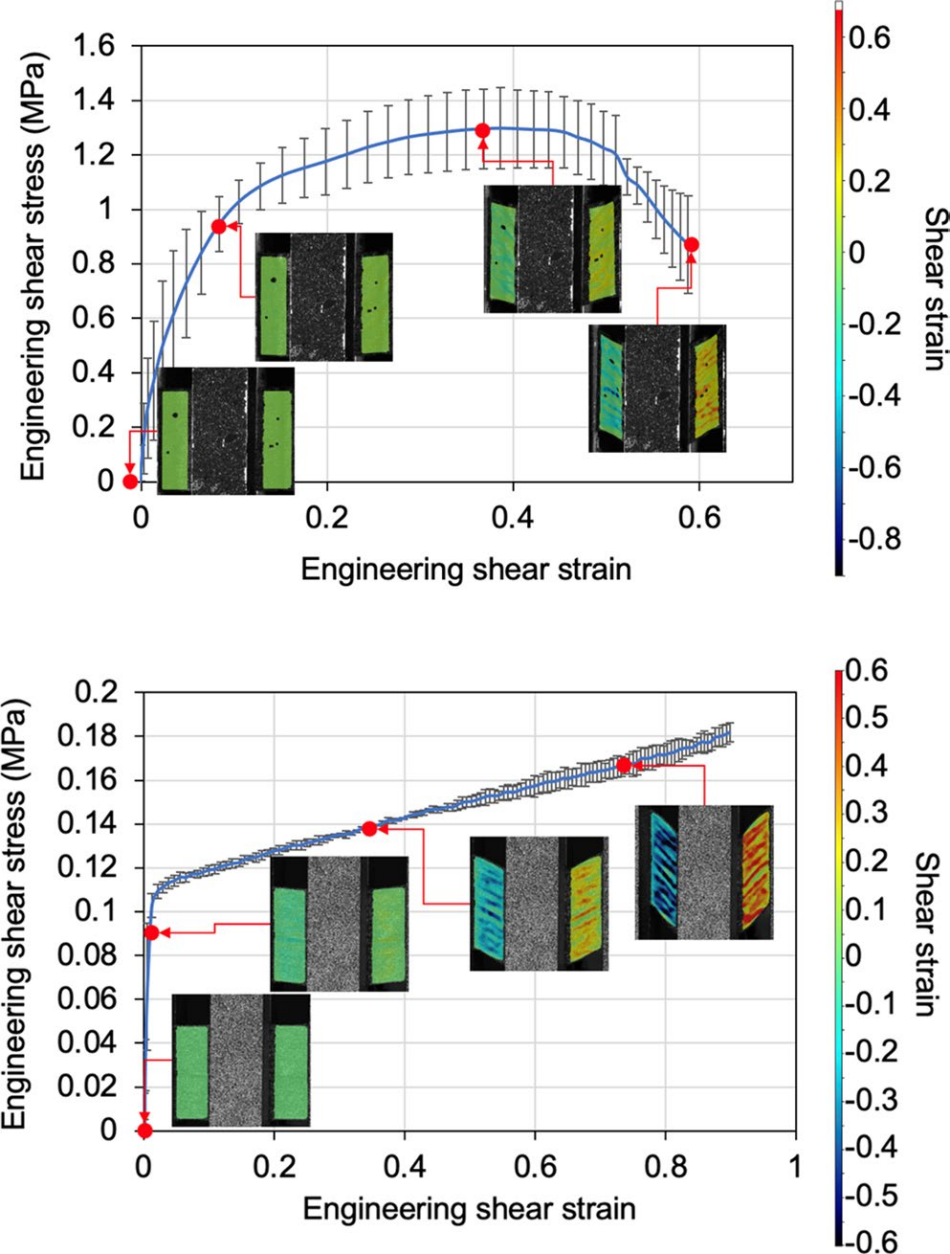
Average shear stress-strain plot of HiPerDiF specimens at (upper) room temperature and (bottom) 50 °C (The subset images show representative DIC images of the sheared regions captured during the modified picture frame test. The grey rectangular region in each subset image is a speckle pattern coated on the middle clamp, which is not relevant to the shear strain measurement)

Figure 10 shows the average engineering shear stress-strain curves of the specimens at room temperature and 50 °C. It shows that the specimens were sheared at a significantly lower shear stress at 50 °C with a small standard deviation, due to the reduced viscosity of the matrix. The shear strain distribution captured by the DIC, as shown in the inset images, indicates that small out-of-plane wrinkles were formed at the early stage, due to the shear buckling. This is consistent with the results reported in the previous work [22].

The tension and shear test results were used as inputs to a bespoke material model for finite element analysis, which is described in “Forming simulation” section.

### Forming experiment

**Fig. 11:**
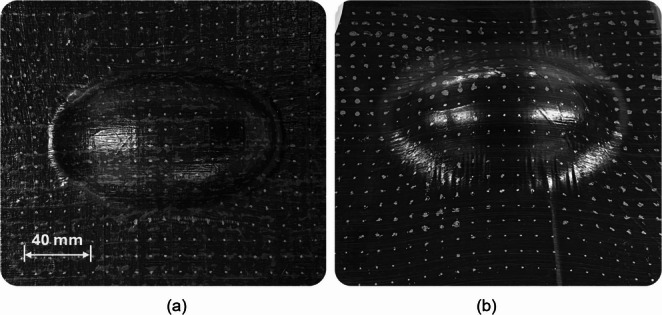
Photos of (**a**) the HiPerDiF preform once formed, and (**b**) the reference continuous fibre preform

Figure 11a shows the images of the preform after forming. The HiPerDiF preform exhibited high conformity to the mould shape, with little to no bridging and minimal wrinkling visible. For a qualitative comparison with the HiPerDiF preform, another [0/90] preform made of a continuous fibre prepreg sheets (MTM49-3/T800, Syensqo, UK) was prepared and formed on the same mould in the exact same condition. In contrast to the discontinuous preform, it is clear from Fig. 11b that this continuous fibre preform could not conform to the mould, particularly around the tight radii between the base plate and the convex surface. Additionally, there were clear signs of fibre bridging and more pronounced wrinkling all around the perimeter of the convex surface.

On detailed inspection of the HiPerDiF preform after forming, some localised thinning of the preform was observed in regions of high deformation, which may have served to aid in its higher degree of conformity. This is a deformation behaviour that cannot be expected from conventional continuous fibre preforms, and such localised thinning or spreading may be classified as a defect when it is excessive in a real component. Such thinning may be more acceptable than fibre bridging, provided it is limited to non-load-bearing areas and remains minimal in extent. Furthermore, it could be compensated for by localised ‘padding up’ or careful design. This ability of the HiPerDiF material to undergo extension emphasises the importance for development of an accurate material model and a robust forming simulation methodology that can take this into account, which is discussed in detail in “Forming simulation” section.

**Fig. 12:**
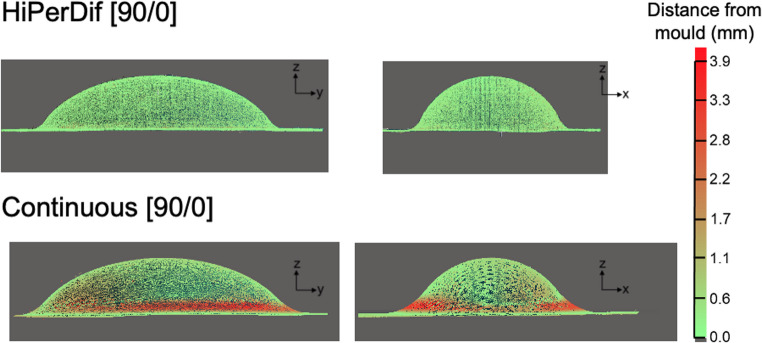
Laser scanned surfaces of the preforms

Laser scan data illustrating the surface conformity of the preform after forming is shown in Fig. 12. The Euclidean distance of the preform from the target mould geometry was quantified using a colour scale, with the high degree of bridging for the continuous preform clearly indicated by the region of red around the perimeter the convex region. The average magnitude of this bridging for the continuous preform is of the order of ~ 4 mm distance from the target geometry, whereas the average magnitude of fibre bridging for the HiPerDiF preform was very low; no deviation above 0.4 mm was detected.

**Fig. 13:**
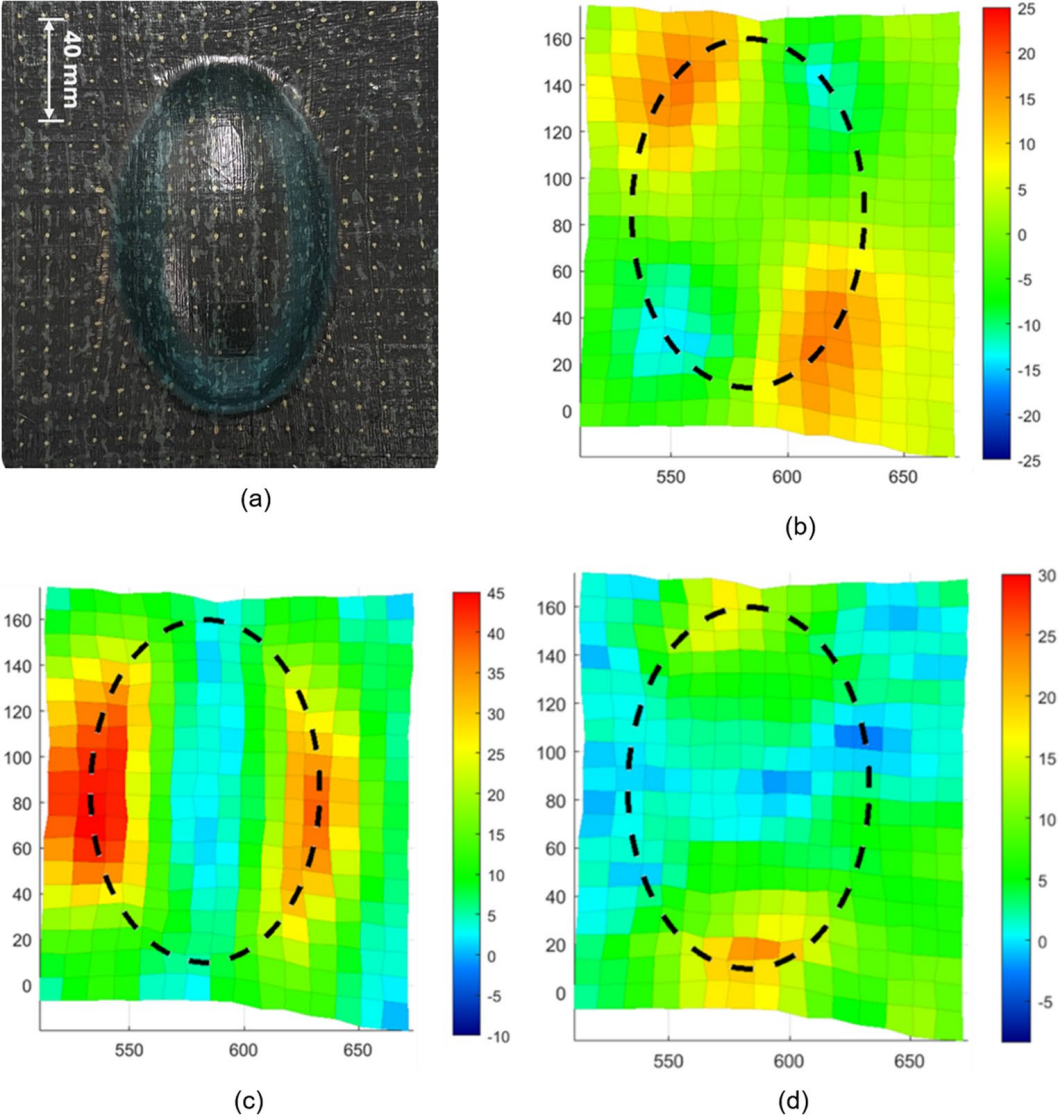
**a** Formed HiPerDiF preform, **b** Shear angle distribution, **c** Axial strain distribution along x-direction, and (**d**) Axial strain distribution along y-direction

The shear angle distribution was calculated using the methods described in “Formability evaluation” section, as shown in Fig. 13b. The colour of each element indicates the average shear angle measured (in degrees) and subsequently smoothed with a mean filter using a window size of 3 by 3 elements. This methodology is commonly used to enhance signal quality, by mitigating random fluctuations without significantly altering the underlying structure of data [23]. As shown in Fig. 13b, the locations of high shear deformation were localised to the four corners of the mould surface – likely principally dominated by a combination of mould curvature and stacking sequence. The measured maximum shear angle values were ~ 18°. It is unlikely that this represents any material-based limit for the HiPerDiF material such as would be expected for continuous materials, due to its higher local extensibility and completely different deformation characteristics.

Axial strain components measured in the local, element-wise directions (as explained in Formability evaluation section) are shown in Fig. 13c and d. The strains were calculated based on the material coordinate system (i.e. local, element-wise). As previously explained, the strain quoted is calculated as the length change of the midlines crossing the element centroid, compared to the reference length of the dot pattern (10 mm).

Despite the cross-ply stacking sequence constraining the transverse spreading of each ply, highly localised axial strains were observed near the corner regions between the doubly-curved surface and the flat substrate. The strain levels reached absolute maximums of around 44%, demonstrating the material’s high formability. The highest strain levels were observed at the locations where the mould’s effective radius is smallest (around the perimeter of the base) and is therefore where bridging for a traditional preform was maximal in this particular DDF setup.

In reference to the material testing results in “Materials characterisation” section, and the observations described above, it is clear that at such high localised strains, the adjacent fibres in the discontinuous material can slide over one another, decreasing the degree of fibre overlap length, and therefore leading to localised thinning. At these regions, the thickness of the component may therefore become too low, leading to a failure during quality control of the part. This is an important behaviour of the discontinuous material that would be very valuable to identify in a numerical model, and it may be the case that end-users forming with such materials require different strategies to address this behaviour, such as local padding up, or optimisation aiming at reducing these areas of excessive tension [24, 25].

### Forming simulation

#### Material model

**Fig. 14:**
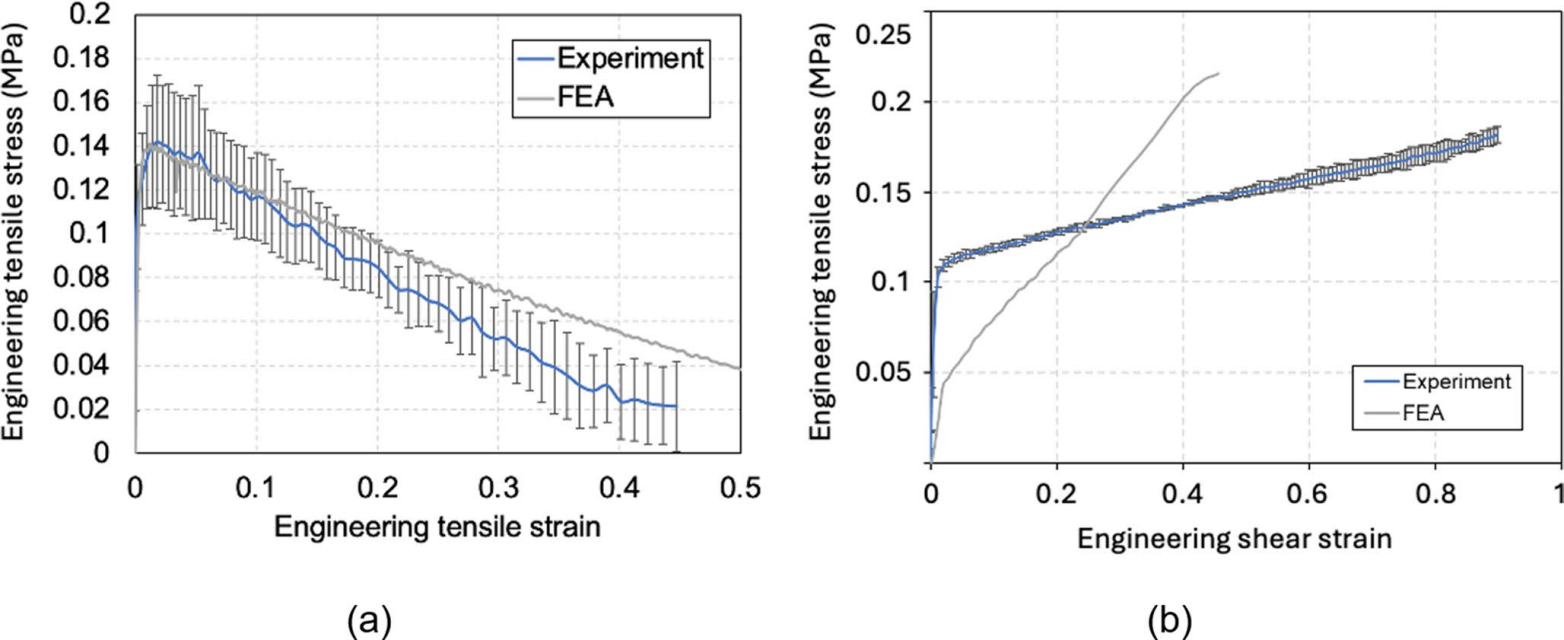
Material responses of the HiPerDiF preform material at 50 °C: **a** tensile stress-strain relationship, and (**b**) in-plane shear stress-strain relationship

The material properties of the HiPerDiF preform obtained by the experiments mentioned in “Materials characterisation” section were also used and the matrix viscosity ($\:\eta\:)$ data was obtained from the material supplier. The storage modulus ($\:G$) was calculated to have the best curve fit to the tensile and shear experimental data demonstrated in Fig. 14. After an acceptable match was obtained in tensile direction, the micromechanical models and properties are tabulated in Table 1. It is important to note that the shear model is still under development. Hence, several limitations affect the interpretation of the shear test results and robust model validation. Firstly, the experimental results were not measured under perfect pure shear conditions due to the presence of fibre tension, which was applied by the test fixture. Secondly, when the stress-strain curve in Fig. 10 reached a plateau, shear-induced wrinkles began to develop in the material. Since tensile deformations were much more dominant than shear deformations, the current shear model [26] was used despite its limited agreement with the experimental results.

**Table 1:** Material properties used for the hybrid element

Element Type	Tensile Properties [Fibre direction]	Tensile Properties [Transverse direction]	Shear Properties	Density [tonne/mm^3^]
Membrane	1E-08 **(MPa)**	1E-08 **(MPa)**	Micromechanical model (Appendix A.1.2.)	7.13E-05
Shell	Micromechanical model (Appendix A.1.1.)	1E-08 **(MPa)**	0 **(MPa)**	7.13E-05

#### Forming simulation results

**Fig. 15:**
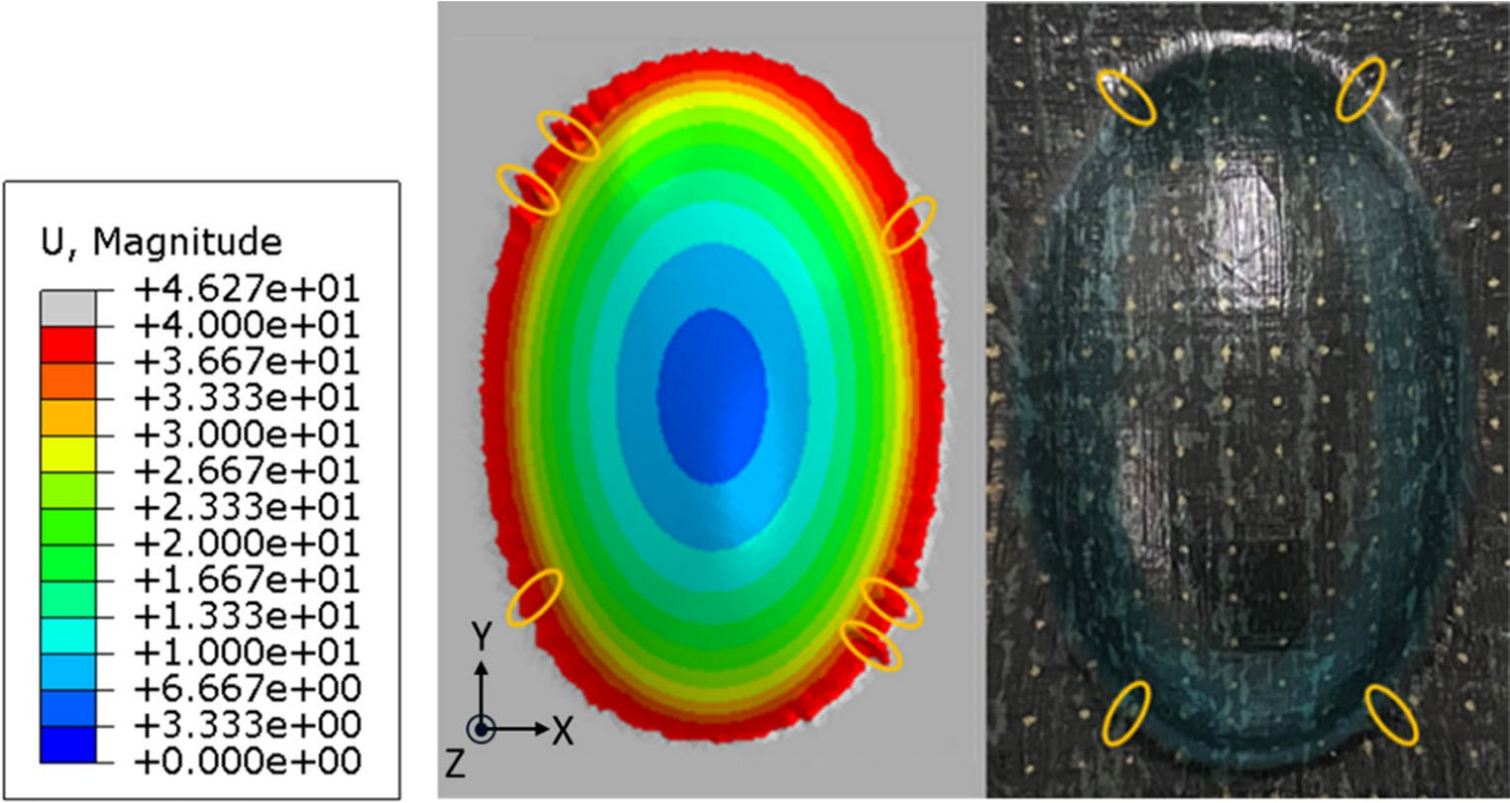
Wrinkles on the HiPerDiF preform after forming: simulation (left) vs. experiment (right)

Globally, the shape of the preform is captured well for both short and continuous fibre specimens, as demonstrated in Fig. 12, which provides a half-cross-sectional view comparing simulations with experimental results. Owing to the high tensile stiffness and low tensile deformability of the continuous fibre specimens, they could not form into the corner of the mould under vacuum pressure. In contrast, the short fibre specimens, characterised by low stiffness and high tensile deformability, easily conformed to the mould’s radii. In Fig. 15, a top-down comparison of the geometry of the formed HiPerDiF preform (right) is shown with the simulation result (left). Localised wrinkling (up to 7.5 mm in length) was observed in the simulation results, as highlighted by the yellow ellipses. Whilst the simulation successfully captured the approximate location of these wrinkles, the absolute magnitudes were slightly more pronounced than those observed in the experimental work. This can be due to several factors: 1- The model inaccuracy in shear (see Fig. 14b); 2- The assumption made on the bending stiffness [27]; 3- The intrinsic variability in the material (see Fig. 9) that can propagate as part-to-part variability [28] and make the validation of deterministic models difficult.

**Fig. 16:**
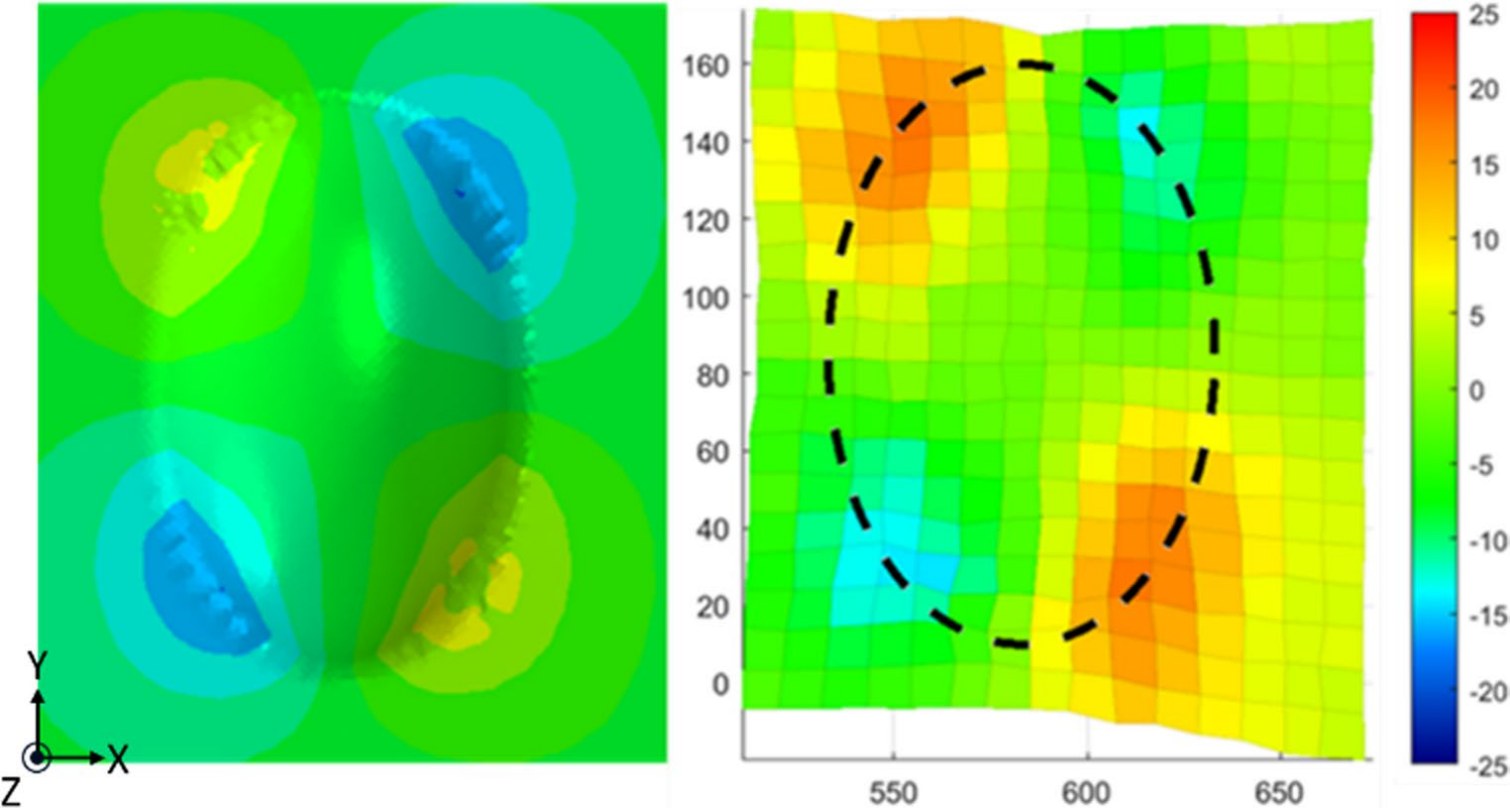
Resultant shear angle deformation around the doubly curved region, post-forming for both the simulation (left) and experimental results (right)

Similarly, the resultant shear angle deformations are shown in Fig. 16, in the simulation (left) and experiments (right). The simulation shows a reasonable agreement in terms of deformation shape and localised regions, although there is a mismatch in magnitude; The maximum shear angle was ± 10.2° for the simulation, whereas the values measured in the experimental work were around + 18°, and − 14°. One of the main reasons for both of these differences could be the mismatch between the model and experimental shear behaviour, shown in Fig. 16. Shear strain in the simulation decreased in areas where wrinkles formed. The presence of more wrinkles in the simulation inhibited further shear deformation, likely due to the increased bending moment in the wrinkled regions, making them more resistant to deformation. Correspondingly, the lower level of shear obtained from the simulation follows from this same property mismatch.

**Fig. 17:**
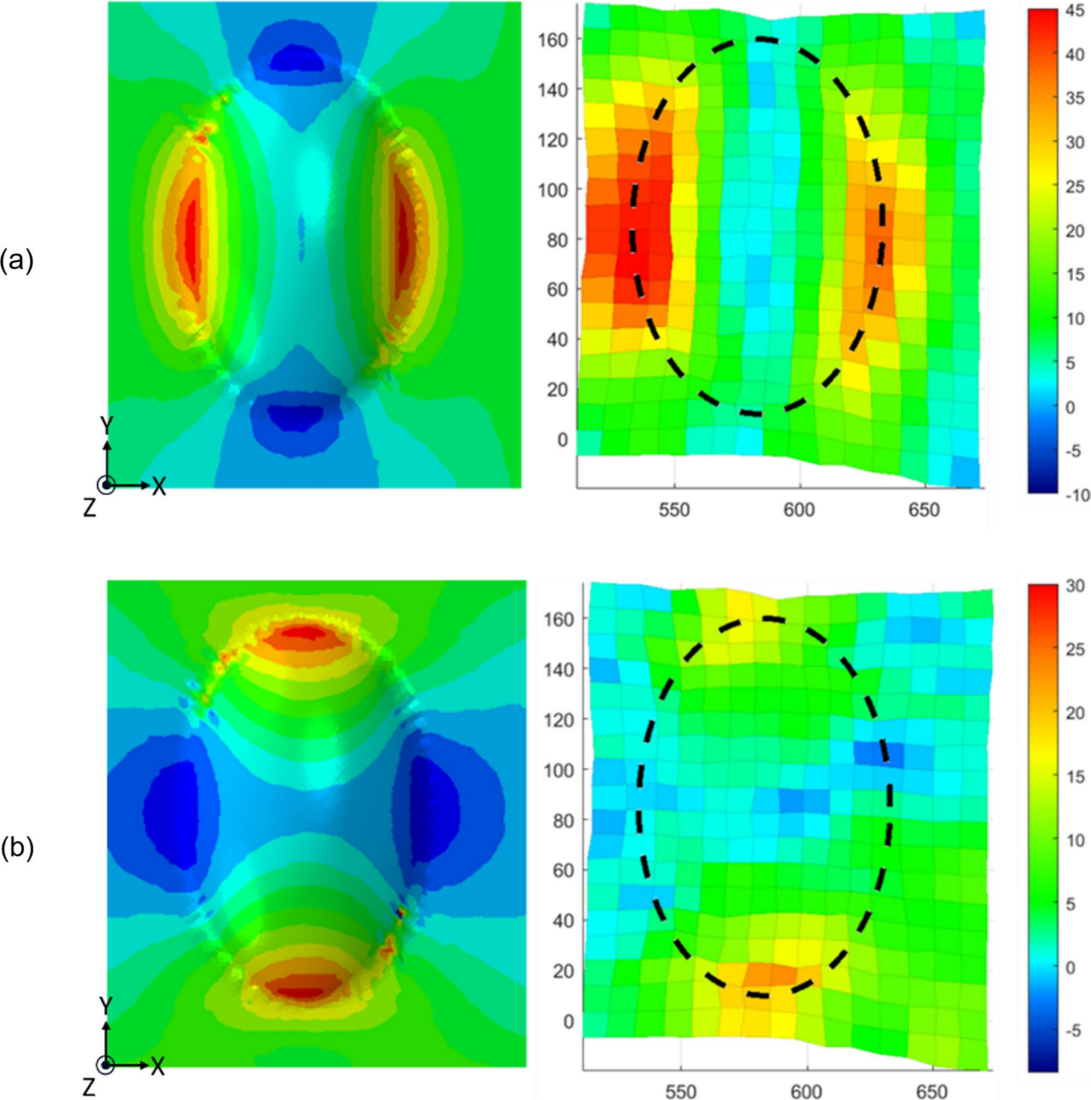
Resultant strain distributions around the doubly curved region, post-forming for both the simulation (left) and experimental results (right): **a** in the x-direction, and (**b**) in the y-direction

The axial strains for both the simulation and experiments are shown in Fig. 17a in the x direction, and Fig. 17b for the y direction. The simulation and experimental results showed good agreement for both directions, with an experimental maximum tensile strain of approximately 40% in the x direction, (taken as an average over both sides shown in red), and a corresponding simulation result of 38%. Similarly, in the y direction the experimental strain of 23% as the average of two localised peak strain points at the top and bottom of the specimen, and the simulation result of 29% tensile strain showed good agreement.

The compressive response of the preform has not been characterised in this study, and therefore the simulation used tensile material properties as proxy compressive properties, by maintaining the same elemental volume. Hence the weaker correlation was expected and could be improved by further investigation in future work.

A key benefit of developing the simulation methodology is the ability to interrogate a given specimen post-forming in ply-by-ply detail without the need for experimental trials. Furthermore, in forming aligned discontinuous fibre materials, the simulation could provide more detailed information that directly impacts the integrity of the formed parts. As seen in the materials characterisation section and the experimental work, the aligned discontinuous fibre materials have the potential to undergo highly localised strains, where adjacent fibres can slide over one another. This decreases the resultant fibre overlap length (ẟ), leading to ply thinning and diminishing the structural integrity of the produced part. In addition, the fibre overlap length (ẟ) determines how much stress can be transferred throughout the ply, as described in Eq. A.1, where a low overlap would lead to a loss of stress transfer between fibres.

**Fig. 18:**
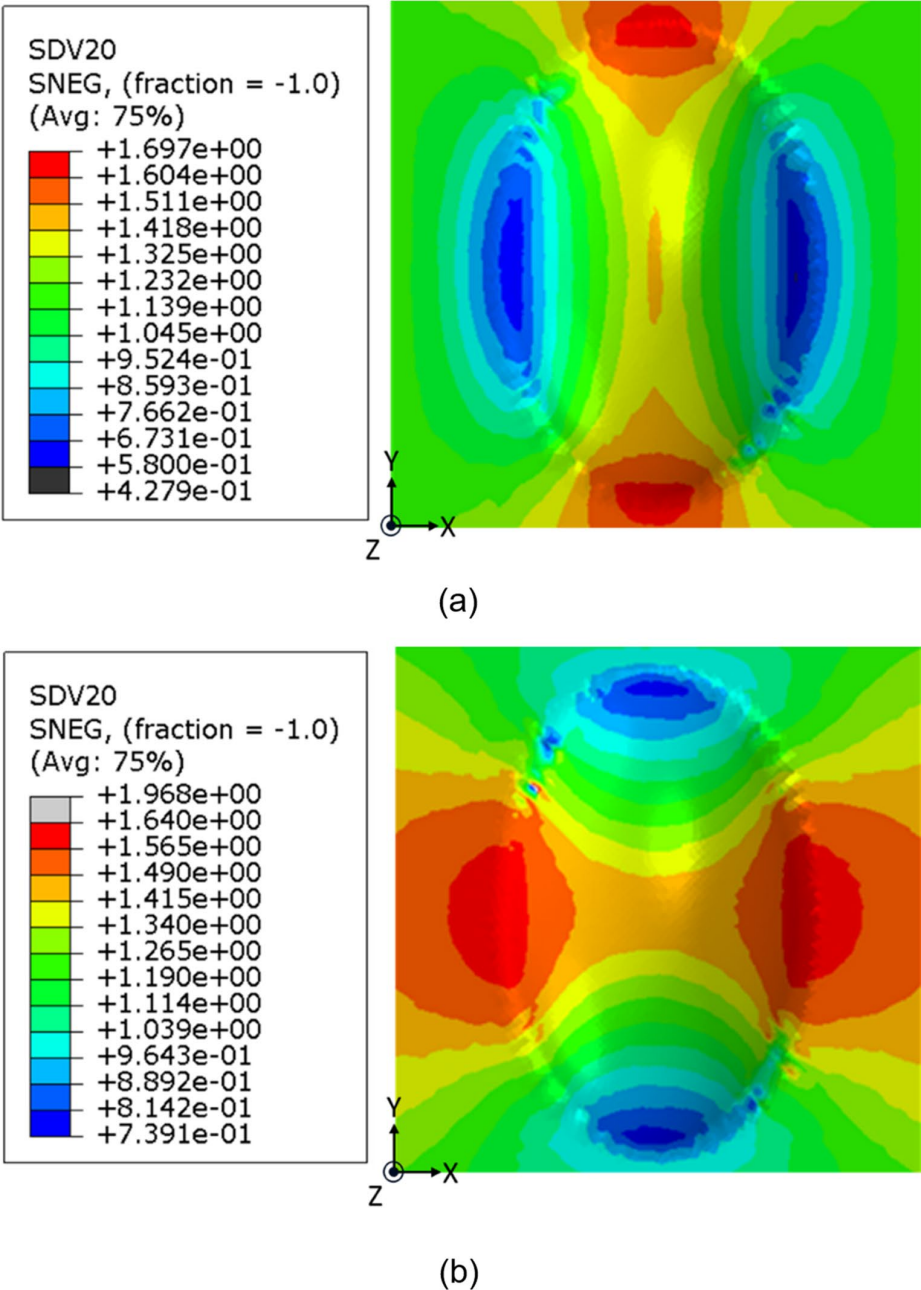
Fibre overlap length distributions around the doubly curved region; (**a**) 0° ply (the first layer), and (**b**) 90° layer (the second layer)

In the current results, the average fibre overlap length was initially assumed to be 1.5 mm, based on the length of the discontinuous fibres used for the HiPerDiF material. The distribution of the fibre overlap length for the 0° and 90° plies after forming, as predicted by the forming simulation, is presented in Fig. 18. In the regions where both plies experience the highest tensile deformation, the fibre overlap length was estimated to decrease from the initial 1.5 mm to a minimum of 0.58 mm. A conservative design estimate for the required minimum fibre overlap length after forming could be obtained from the critical fibre length for epoxy and carbon fibre, which can be calculated as 0.4 mm, assuming an interfacial shear strength of 35 MPa and a fibre strength of 4100 MPa [29, 30]. As a result, given that the total fibre lengths are considerably greater than this critical length and the fibre overlap length also exceeds this threshold, the resultant stress transfer in the discontinuous plies is likely to be sufficient, and the obtained results could be considered a safe level of deformation. Further experimental studies to validate this approach would be of great benefit to end-users.

Furthermore, to account for ply thinning associated with the reduction in fibre overlap length, the thickness can be calculated under the assumption of the volume of element is constant. Given the resultant fibre overlap length is 0.58 mm, the layer thickness is estimated to decrease by approximately 61%, thereby potentially creating a weak region in the structure, depending on intended use. This simulation methodology allows to capture the evolution of fibre overlap length, which is directly linked to preform tearing as it approaches zero. Additionally, the final thickness of the formed part can be reliably estimated based on this overlap behaviour.

## Conclusions

The formability of aligned discontinuous fibre composite preforms manufactured using the HiPerDiF method has been studied both numerically and experimentally, with qualitative and quantitative comparisons. The HiPerDiF preform exhibited superior conformity to the target doubly curved geometry compared to the continuous fibre preform in double diaphragm forming. Fibre bridging was effectively avoided due to the high stretchability along the fibre direction. This improved formability was shown to largely result from localised axial strain being much greater in the HiPerDiF preform, reaching maximum values of up to 40%.

A previously developed material model and accompanying forming simulation methodology were used to capture the forming behaviour of the HiPerDiF preform. It is, however, worth noting that the variability inherent in the material system limits the degree of conformity between model and experiment; Nevertheless, the simulation still provides valuable insight into the deformation mechanisms and could serve as a useful tool for optimising forming conditions. It is in particular able to give an estimation the deformation of the layers inside the laminate which cannot be measured experimentally and is useful for examining high-strain regions and identifying any associated ply thinning or reduced fibre overlap length, which could impact compliance with design requirements.

This study has demonstrated the advantage of aligned discontinuous fibre materials over continuous fibre materials in enhancing manufacturability and reducing defects in the production of complex composite parts, while also identifying key research areas necessary to establish a framework for designing such components, including the development of reliable preform tearing criteria and the mitigation of material thinning.

## Data Availability

No datasets were generated or analysed during the current study.

## Appendix

### Rate equations

Hypo-elastic and hypo-viscoelastic models, which are constitutive models that incorporate rate-dependent behaviour, represent one of the prevailing methodologies for characterising the mechanical response of fibrous sheets, whether dry or impregnated, within a two-dimensional continuum mechanics framework. The alternative approach commonly employed is the hyper-viscoelastic method, as delineated in prior literature. The fundamental premise of this approach involves iteratively capturing the material’s nonlinear response through incremental steps and establishing the relationship between stress and strain at each step. The general formulation of these models is articulated as follows:

A.1 $$\:{\widehat{\boldsymbol{\sigma\:}}}_{ij}=\boldsymbol{C}:{\dot{\boldsymbol{\varepsilon\:}}}_{ij}$$

where $\:{\boldsymbol{\sigma\:}}_{ij}$ represents the Eulerian Cauchy stress tensor and $\:{\dot{\boldsymbol{\varepsilon\:}}}_{ij}$ denotes the strain rate tensor. The constitutive tensor $\:\boldsymbol{C}$ is employed to delineate the material’s behaviour. Owing to the lack of frame indifference or objectivity in the derivative of $\:{\boldsymbol{\sigma\:}}_{ij}$ (i.e., its invariants are contingent upon the chosen coordinate system), Eq. A.1 is instead articulated in relation to the objective stress rate, $\:{\widehat{\boldsymbol{\sigma\:}}}_{ij}$. This objective stress rate, $\:{\widehat{\boldsymbol{\sigma\:}}}_{ij}$, is connected to $\:{\boldsymbol{\sigma\:}}_{ij}$ and the rigid body rotation, $\:\boldsymbol{Q}$, as outlined in the following equation.

A.2 $$\:{\widehat{\boldsymbol{\sigma\:}}}_{ij}=\boldsymbol{Q}\left[\frac{d}{dt}\left({\boldsymbol{Q}}^{T}{\boldsymbol{\sigma\:}}_{ij}\boldsymbol{Q}\right)\right]{\boldsymbol{Q}}^{T}$$

In conventional Finite Element (FE) implementations, objective stress rates are typically computed using either the Green-Naghdi (G-N) or Jaumann frameworks, and the rotation tensor $\:\boldsymbol{Q}$ is derived based on either polar or corotational frame rotation. However, as highlighted by Badel et al. [31], due to material anisotropy and the presence of fibres, the rotation of the loading direction differs from that of the global coordinate system. Consequently, the objective stress rate, $\:{\widehat{\boldsymbol{\sigma\:}}}_{ij}$, should be expressed with respect to the fibre direction, necessitating a corresponding transformation of both strain and stress tensors. Given that Abaqus/Explicit expects stress calculations based on the GN objective rate, procedures must be devised to rotate various stress and strain quantities (along with their respective rates) from the global coordinate system, {$\:{\boldsymbol{e}}_{i}$}, to the fibre frame, {$\:{\boldsymbol{f}}_{i}$}, where i = 1,2. By convention, $\:{f}_{1}$ aligns with the fibre direction (in the case of a fabric, $\:{f}_{2}\:$would align with the second fibre direction). Following deformation, the global coordinate system and fibre frames can be represented as follows:

A.3 $$\:{\boldsymbol{e}}_{i}=\boldsymbol{Q}{\boldsymbol{e}}_{i}^{0}$$

A.4 $$\:{\boldsymbol{f}}_{i}=\frac{\boldsymbol{F}{\boldsymbol{f}}_{i}^{0}}{\parallel \boldsymbol{F}{\boldsymbol{f}}_{i}^{0}\parallel}$$

where $\:{\boldsymbol{e}}_{i}^{0}$ and $\:{\boldsymbol{f}}_{i}^{0}\:$represent the initial direction vectors of the global coordinate system and the fibre frame, respectively, and $\:\boldsymbol{F}$ denotes the material’s deformation gradient. $\:\boldsymbol{Q}$ denotes the rotation frame derived from the polar decomposition of $\:\boldsymbol{F}$, as outlined in Eq. A.5.

A.5 $$\:\boldsymbol{F}=\boldsymbol{Q}\boldsymbol{U}$$

In commercial FE software, user material subroutines provide both the deformation gradient tensor, $\:\boldsymbol{F}$, and the stretch tensor, $\:\:\boldsymbol{U}$, at the initiation of each increment. Consequently, the rotation tensor, $\:\boldsymbol{Q}$, can be readily computed by rearranging Eq. A.5. Within the Green-Naghdi (G-N) framework, the angular velocity of the corresponding rigid body rotation, denoted as $\:\varOmega\:=\dot{\boldsymbol{Q}}\cdot\:{\boldsymbol{Q}}^{T}$, is utilised, enabling the expression of the objective Cauchy stress derivative as follows:

A.6 $$\:{\widehat{\boldsymbol{\sigma\:}}}_{ij}=\dot{\boldsymbol{\sigma\:}}-\varOmega\:\boldsymbol{\sigma\:}-\boldsymbol{\sigma\:}{\varOmega\:}^{T}$$

The angle between the fibre directions and the directions of the global coordinate system can be computed as presented in Eq. A.7. Subsequently, the transformation matrix connecting {$\:{\boldsymbol{e}}_{i}$} to {$\:{\boldsymbol{f}}_{i}$} can be derived directly from this angle, as expressed in Eq. A.8.

A.7 $$\:{\theta\:}_{i}={\mathrm{tan}}^{-1}\left(\frac{\parallel {\boldsymbol{e}}_{i}\times\:{\boldsymbol{f}}_{i}\parallel}{\parallel {\boldsymbol{e}}_{i}{\boldsymbol{f}}_{i}\parallel}\right)$$

A.8 $$\:{\boldsymbol{T}}^{\left(\boldsymbol{i}\right)}=\left[\begin{array}{cc}\mathrm{cos}{\theta\:}_{i}&\:-\mathrm{sin}{\theta\:}_{i}\\\:\mathrm{sin}{\theta\:}_{i}&\:\mathrm{cos}{\theta\:}_{i}\end{array}\right]$$

The transformation matrices described in Eq. A.8 are subsequently utilised to project (refer to Eq. A.9) the strain increment tensor onto each direction of the fibre frame {$\:{\boldsymbol{f}}_{i}$}, employing the strain increment in the global coordinate system {$\:{\boldsymbol{e}}_{i}$}. This information is typically supplied to any user material subroutine for commercial FE codes.

A.9 $$\:{{d\boldsymbol{\epsilon\:}}_{f}}^{\left(i\right)}={\boldsymbol{T}}^{\left(i\right)}{d\boldsymbol{\varepsilon\:}}_{e}{{\boldsymbol{T}}^{\left(i\right)}}^{T}$$

Following this procedure, the constitutive equations (as per Eq. A.1) can be invoked to derive the corresponding stress increment component linked with each direction of the fibre frame.

A.10 $$\:{{d\boldsymbol{\sigma\:}}_{ij}}^{\left(i\right)}={\boldsymbol{C}}_{f}{{d\boldsymbol{\varepsilon\:}}_{ij}}^{\left(i\right)}$$

The stress state on each fibre direction at increments of *n* + 1 can be determined by integrating Eq. A.11 utilising a mid-point rule.

A.11 $$\:{{{\boldsymbol{\sigma\:}}^{n+1}}_{f}}^{\left(i\right)}={{{\boldsymbol{\sigma\:}}^{n+1}}_{f}}^{\left(i\right)}+{{\boldsymbol{C}}^{n+1/2}}_{f}{{d{\boldsymbol{\varepsilon\:}}^{n+1/2}}_{f}}^{\left(i\right)}$$

The final step in the hypo-viscoelastic continuum depiction of fibrous materials involves rotating the stress tensors associated with each direction of the fibre frame back into the global coordinate system and consolidating the total stress tensor of the material from the resultant tensors, as outlined in Eq. A.12.

A.12 $$\:{\boldsymbol{\sigma\:}}^{n+1}=\sum\:_{\boldsymbol{i}=1}^{2}{{\boldsymbol{T}}^{\left(i\right)}}^{T}{{{\boldsymbol{\sigma\:}}^{n+1}}_{f}}^{\left(i\right)}{\boldsymbol{T}}^{\left(i\right)}$$

### Tensile Implementation

The tensile analytical micromechanical model has been integrated in the framework outlined in the preceding section through the utilisation of a material subroutine for the (FE) package Abaqus/Explicit. The matrix shear strain rate ($\:{\dot{\gamma\:}}_{m}$) is calculated by using Eq. A.13 where fibre length (*L*) = 3 mm, fibre diameter (𝐷) = 7 μm, fibre volume fraction (𝑓) = 0.27, the strain rate in the fibre direction ($\:{\dot{\epsilon\:}}_{11}$) and the parameter K = 2.64 is a geometric factor used to calculate the shear deformation within the matrix, based on the assumption of a hexagonal fibre packing arrangement. It arises from the relation K = S/(S-D), where D is the fibre diameter and S is the centre-to-centre distance between adjacent fibres [26]. Then, the storage modulus ($\:G$) and the viscosity ($\:\eta\:$) are used within Eq. A.14 to calculate the change in matrix shear stress ($\:\varDelta\:{\tau\:}_{m}$) at that time step. Finally, the stress change in the fibre direction ($\:\varDelta\:{\sigma\:}_{11}$) is calculated as in Eq. A.15 and implemented into Eq. A.10 as the stress change in the fibre direction. The overlap length ($\delta$) was calculated as a function of tensile strain which is the Eq. A.16.

A.13 $$\:\dot{{\gamma\:}_{m}}=\frac{L-\delta}{D}\left[K-1\right]{\dot{\varepsilon\:}}_{11}$$

A.14 $$\:\varDelta\:{\tau\:}_{m}=\varDelta\:t\left(\eta\:{\dot{\gamma\:}}_{m}-{\tau\:}_{m}\right)\frac{G}{\eta\:}$$

A.15 $$\:\varDelta\:{\sigma\:}_{11}=2\varDelta\:{\tau\:}_{m}f\frac{\delta}{D}$$

A.16 $${\delta}=\frac{L}{2}(1.0101{\epsilon\:}^{2}-2.0101\varepsilon\:+1)$$

### Shear Implementation

The shear analytical micromechanical model was integrated into the existing framework *via* a custom user material subroutine (VUMAT) for the FE package Abaqus/Explicit. The matrix shear strain rate ($\:{\dot{\gamma\:}}_{m}$) is first calculated using Eq. A.17 and the in-plane shear strain rate ($\:{\dot{\epsilon\:}}_{12}$). The storage modulus ($\:G$) and the viscosity ($\:\eta\:)$ are then used in Eq. A.18 to determine the change in matrix shear stress ($\:\varDelta\:{\tau\:}_{m}$) at a given time increment. Finally, the corresponding change in in-plane shear stress ($\:\varDelta\:{\sigma\:}_{12}$) is calculated using Eq. A.19 and incorporated into the general stress update procedure described in Eq. A.10:

A.17 $$\:\dot{{\gamma\:}_{m}}=\frac{S}{S-D}{\dot{\epsilon\:}}_{12}$$

A.18 $$\:\varDelta\:{\tau\:}_{m}=\varDelta\:t\left(\eta\:{\dot{\gamma\:}}_{m}-{\tau\:}_{m}\right)\frac{G}{\eta\:}$$

A.19 $$\:\varDelta\:{\sigma\:}_{12}=\:\varDelta\:{\tau\:}_{m}$$

## Footnotes

1 HiPerDiF technology was developed at the University of Bristol through funding from the Engineering and Physical Sciences Research Council (EP/I02946X/1 and EP/P027393/1) and later commercialised as AFFT™ (aligned formable fibre technology) by the University of Bristol spin out company, Lineat Composites Ltd.
